# Supplementary figures and images for: Atmospheric Reaction Systems as Null-Models to Identify Structural Traces of Evolution in Metabolism
Source: PLoS One. 2011 May 6;6(5):e19759. doi: 10.1371/journal.pone.0019759 (PMC3089637; doi:10.1371/journal.pone.0019759)

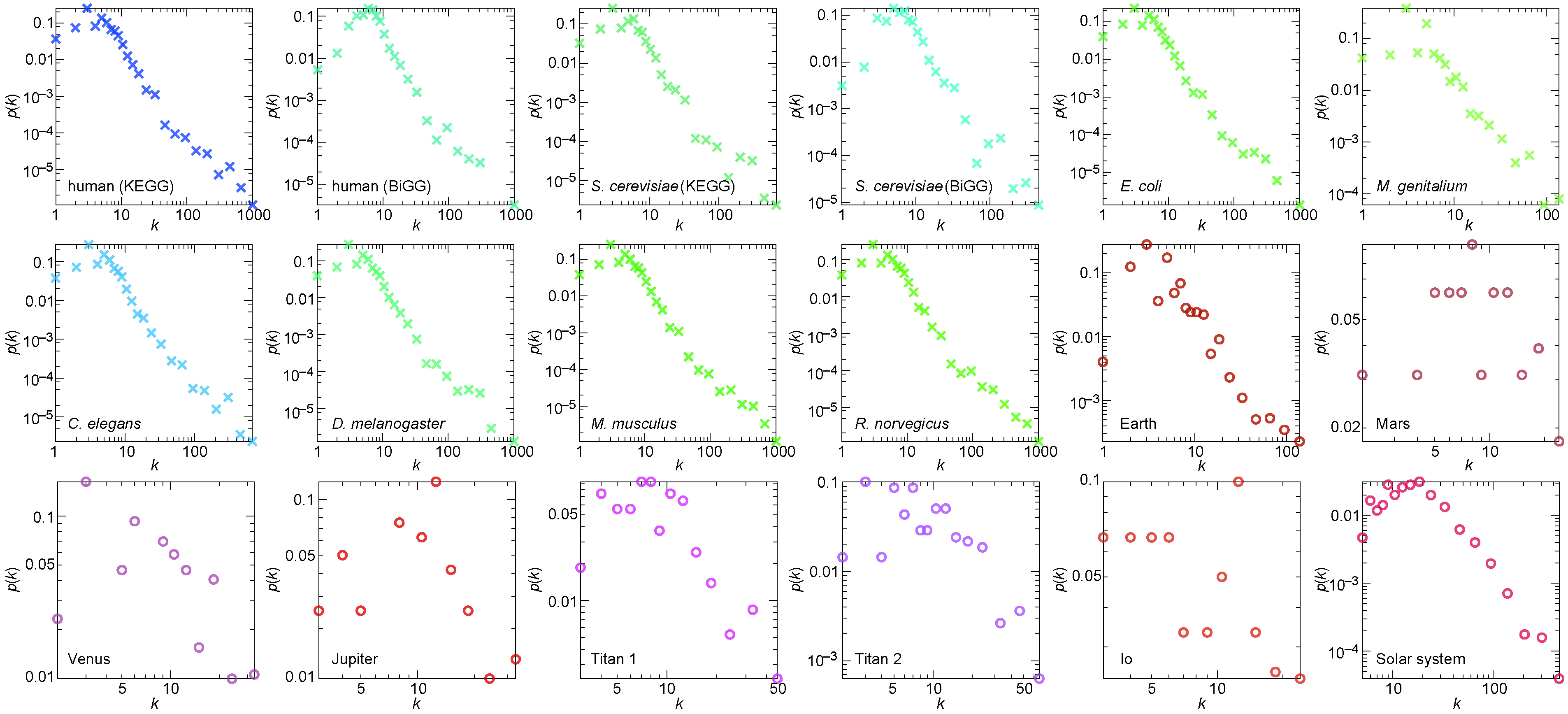

Supplement: Figure S1 — Degree distributions for the substance networks. The data is log-binned and plotted in log–log scale. (TIF) [file pone.0019759.s001.tif]

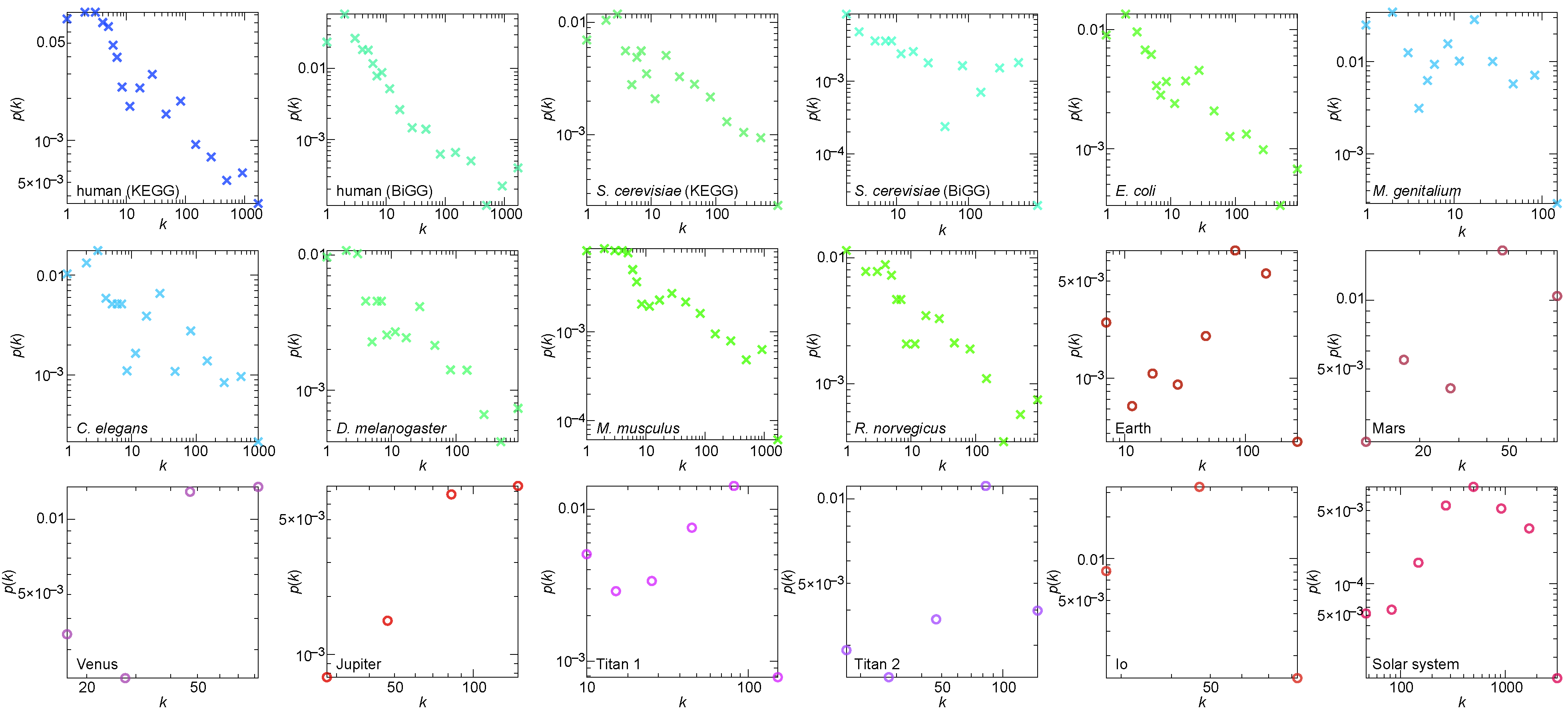

Supplement: Figure S2 — Degree distributions for the reaction networks. The data is log-binned and plotted in log–log scale. (TIF) [file pone.0019759.s002.tif]

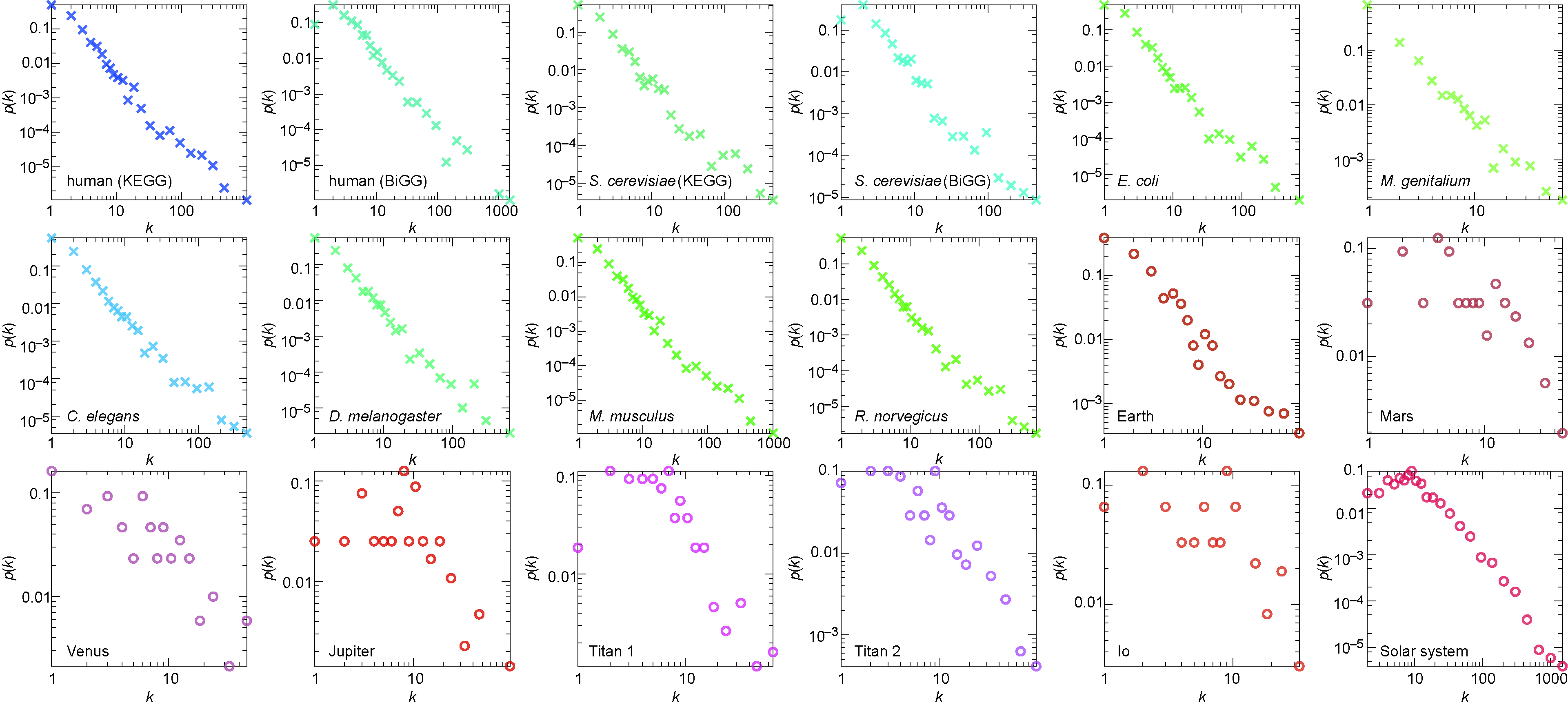

Supplement: Figure S3 — Degree distributions for the substances in the bipartite representations. The data is log-binned and plotted in log–log scale. (TIF) [file pone.0019759.s003.tif]

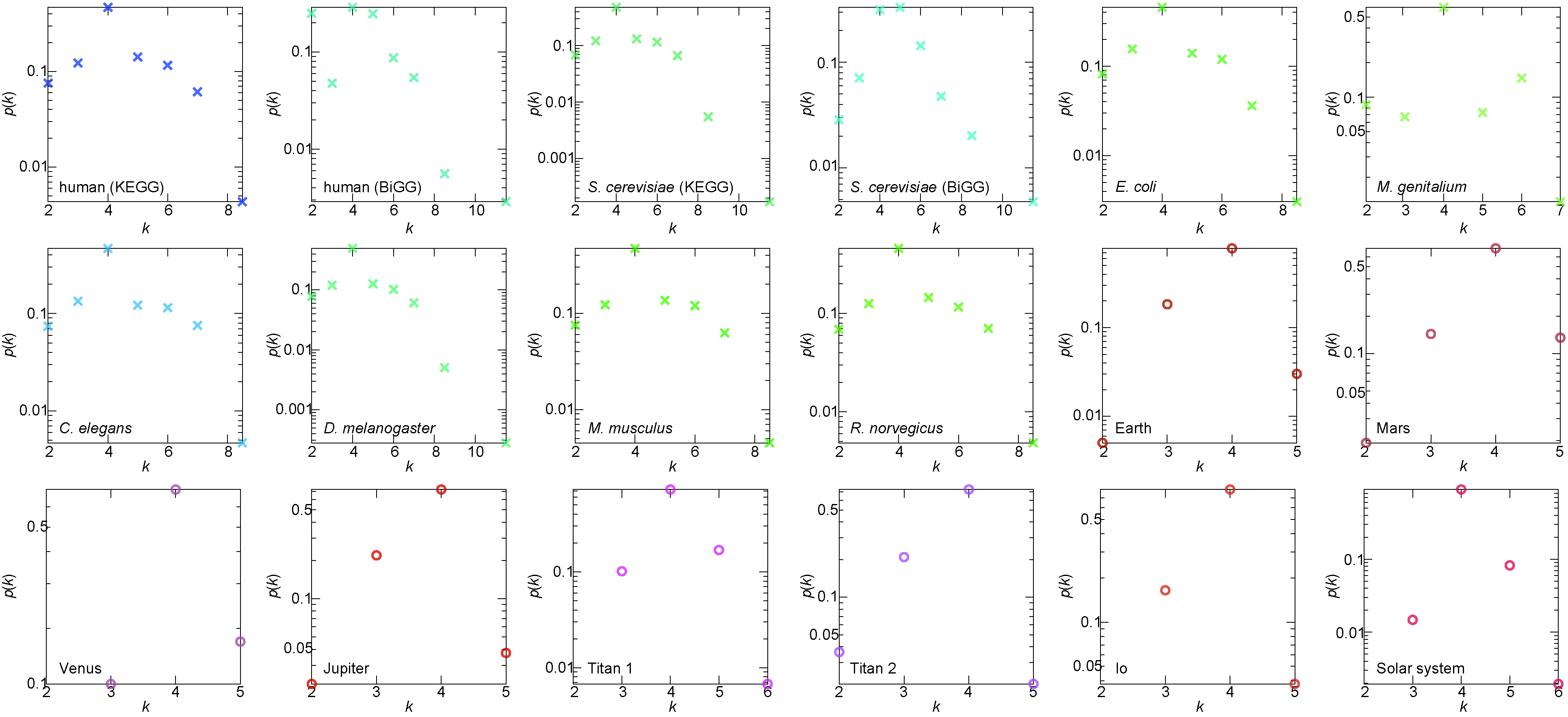

Supplement: Figure S4 — Degree distributions for the reactions in the bipartite representations. The data is log-binned and plotted in log–log scale. (TIF) [file pone.0019759.s004.tif]

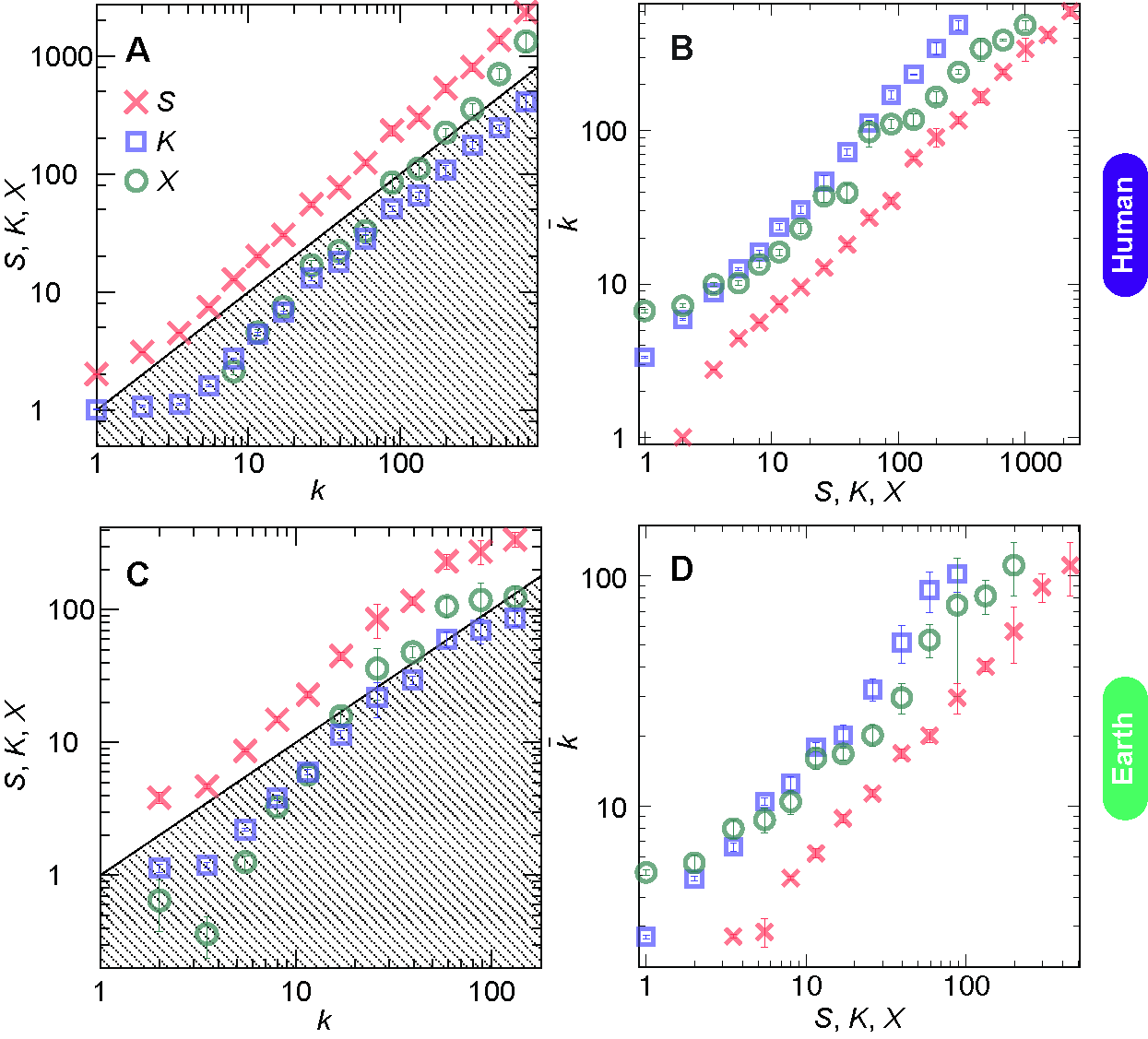

Supplement: Figure S5 — A plot corresponding to Fig. 3C, D, G and H for substance networks. Panels A and C display the values of the three terms of k—S, K and X. The diagonal line shows the k-value. Panels B and D show the average degrees of nodes with certain values of the three terms that contribute to the degree in the projected networks. is averaged over logarithmic bins of S, K, and X values. Panels A and B is data for the human network; C and D are the corresponding plots for the Earth atmospheric network. (TIF) [file pone.0019759.s005.tif]
